# Methylprednisolone inhibits interleukin-17 and interferon-gamma expression by both naive and primed T cells

**DOI:** 10.1186/1471-2172-9-47

**Published:** 2008-08-12

**Authors:** Miljana Momčilović, Željka Miljković, Dušan Popadić, Miloš Marković, Emina Savić, Zorica Ramić, Djordje Miljković, Marija Mostarica-Stojković

**Affiliations:** 1 Department of Immunology, Institute for Biological Research "Siniša Stanković", Belgrade, Serbia; 2 Institute of Microbiology and Immunology, School of Medicine, University of Belgrade, Belgrade, Serbia

## Abstract

**Background:**

Interleukin-17 (IL-17)-producing cells are increasingly considered to be the major pathogenic population in various autoimmune disorders. The effects of glucocorticoids, widely used as therapeutics for inflammatory and autoimmune disorders, on IL-17 generation have not been thoroughly investigated so far. Therefore, we have explored the influence of methylprednisolone (MP) on IL-17 expression in rat lymphocytes, and compared it to the effect of the drug on interferon (IFN)-γ.

**Results:**

Production of IL-17 in mitogen-stimulated lymph node cells (LNC) from non-treated rats, as well as in myelin basic protein (MBP)-stimulated draining LNC from rats immunized with spinal cord homogenate and complete Freund's adjuvant was significantly reduced by MP. The reduction was dose-dependent, sustained through the follow-up period of 48 hours, and was not achieved through anti-proliferative effect. Additionally, MP inhibited IL-17 production in purified T cells as well, but to less extent than in LNC. In its influence on IL-17 production MP inhibited Ror-γT transcription factor expression, as well as Jun phosphorylation, but not ERK or p38 activation in mitogen-stimulated LNC. Importantly, MP collaborated with IFN-γ in inhibiting IL-17 generation in LNC.

**Conclusion:**

The observed difference in the effect of MP on IL-17 and IFN-γ could be important for the understanding of the variability in the efficiency of glucocorticoids in the treatment of autoimmune diseases.

## Background

Interleukin-17 (IL-17A or IL-17) is the prototypic member of a newly identified cytokine family which comprises five other relatives: IL-17B-F [1]. This cytokine exerts its pleiotropic effects by binding to the IL-17 receptor with ubiquitous tissue and cell distribution. It promotes inflammation through enhancing the production of diverse pro-inflammatory cytokines and mediators, including IL-6, IL-8, G-CSF, leukemia inhibitory factor, PGE2, nitric oxide, as well as proliferation, maturation and chemotaxis of neutrophiles [2]. It is mainly produced by effector and memory CD4^+ ^T lymphocytes developed from a unique lineage of CD4^+ ^T cells distinct from Th1 and Th2 effectors, and negatively regulated by their respective signature cytokines IFN-γ and IL-4 [3,4]. These newly described effectors – Th17 cells, at least in mice, develop from naïve CD4^+ ^T cells under the influence of TGF-β and IL-6 [5-7], require IL-23 for survival and expansion [8], and secrete a profile of inflammatory cytokines including IL-17 and IL-17F, IL-6, GM-CSF, TNF-α, IL-21 and IL-22 [2]. IL-17 has emerged as a crucial pathogenic factor in several autoimmune and inflammatory diseases induced in experimental animals, such as experimental autoimmune encephalomyelitis (EAE), collagen induced arthritis (CIA), inflammatory bowel disease (IBD), previously thought to be mediated by Th1 cells [9]. Additionally, dysregulation of IL-17 production was found to be associated with many chronic inflammatory diseases in humans, such as rheumatoid arthritis (RA), asthma, IBD, multiple sclerosis (MS), psoriasis vulgaris, as well as with allograft rejection [10].

Glucocorticoids (GCs) are steroid hormones that are among the most potent immunosuppressive and anti-inflammatory drugs currently available. Synthetic GCs are efficacious in the treatment of numerous inflammatory and autoimmune diseases and in preventing graft rejection, while endogenously produced GCs play an essential and complex role in the regulation of the immune response [11]. They have been shown to affect both innate and adaptive immune response by influencing cell trafficking, proliferation, expression of surface molecules, such as MHC, co-stimulatory and adhesion molecules, and synthesis of many inflammatory mediators, including cytokines [11]. GCs exert most, if not all, of their effects through binding to the glucocorticoid receptor (GR), a ligand-activated transcription factor [12,13]. Their influence on T cell functions is both direct and indirect, via antigen-presenting cells (APCs). It is known that GC-GR complexes inhibit both T cells and APCs functions by affecting key transcription factors involved in the regulation of expression of a number of inflammatory cytokines, such as IFN-γ, TNF-α and IL-2 [14]. Additionally, several studies clearly showed both in animals and humans that the presence of GCs enhanced Th-2 cytokines IL-4, IL-10 and IL-13 at the same time as they decreased Th-1 cytokines secretion by CD4^+ ^lymphocytes [15,16].

Since compelling evidence indicate that GCs differentially regulate the production of Th1 and Th2 cytokines, it is important to know whether and if so, how GCs affect the production of IL-17, a cytokine accused to be critically involved in the pathogenesis of autoimmune and chronic inflammatory diseases frequently treated with GCs. Therefore, the aim of this study was to analyze *in vitro *effects of methylprednisolone (MP), a synthetic glucocorticoid drug, on mitogen- and antigen-induced expression and production of IL-17 in the rat and to compare these effects to corresponding effects on IFN-γ. As the result, we show that MP inhibits mitogen- and antigen-induced IL-17 expression, but less potently than corresponding IFN-γ expression.

## Methods

### Experimental animals

Inbred Dark Agouti (DA) and Albino Oxford (AO) rats were obtained from animal breeding facility of the Institute for Biological Research "Siniša Stanković" (Belgrade) and were kept under standardized conditions. Age- and gender-matched animals, 12–16 weeks old, were used for experiments. Rats were housed under conventional conditions with laboratory chow and water *ad lib*. For the experiments investigating antigen-specific production of cytokines, DA rats were immunized with mixture of rat spinal cord homogenate and complete Freund's adjuvant (SCH-CFA), as described previously [17]. All experiments were approved by the Ethical Committee of the Institute for Biological Research "Siniša Stanković" (IBISS, N° 16/07).

### Chemicals, cells and cell cultures

Methylprednisolone (MP) was from Hemofarm (Vršac, Serbia), concanavalin A (ConA) was from Pharmacia (Uppsala, Sweden), guinea pig myelin basic protein (MBP) was a kind gift of Dr Alexander Fluegel (Max-Planck Institute for Neurobiology, Martinsried, Germany). ERK-inhibitor UO126, p38-inhibitor SB202190 and Jnk-inhibitor SP600125 were from Sigma (Deisenhofen, Germany). Neutralizing anti-IFN-γ antibody and isotype control antibody of irrelevant specificity were from Holland Biotechnology (Leiden, The Netherlands). Cervical, popliteal, inguinal and para-aortal lymph node cells (LNC) were isolated from healthy animals, and draining popliteal lymph node cells (DLNC) and cells infiltrating spinal cord (SCC) from DA rats immunized with SCH-CFA, as described previously [17]. All the cells were grown at 5% CO_2 _and 37°C in RPMI-1640 (Sigma) supplemented with antibiotics and 5% fetal calf serum (PAA Laboratories, Pasching, Austria) for LNC or 2% rat serum for DLNC and SCC. LNC were stimulated with ConA (2.5 μg/ml) and were seeded in 96-well plates for proliferation assay (2 × 10^5 ^cells/200 μl) or in 24-well plates (2 × 10^6^/ml) for determination of cytokines. DLNC and SCC were seeded in 96-well plates (5 × 10^5^/200μl) and stimulated with MBP (10 μg/ml). For the purification of T cells, anti- rat CD3-biotin conjugated antibody (BD Biosciences, San Diego, CA) and MACS streptavidin microbeads and MACS separation columns were used according to the instructions of the manufacturer (Miltenyi Biotec, Aubum, CA). The obtained cells were more than 98% positive for CD4 or CD8 as deduced by cytofluorometry (FACS Calibur, BD Biosciences), and were stimulated with plate bound anti-CD3 (1 μg/ml) and anti CD28 (1 μg/ml) antibodies (eBioscience, San Diego, CA). The population of CD3^- ^cells, obtained by the same procedure as cells that were not bound to CD3- biotin conjugated antibody which were more than 98% negative for CD3 (as deduced by cytofluorimetry), were stimulated with LPS (1 μg/ml, Sigma).

### Reverse transcription- real time polymerase chain reaction

In order to determine cytokine? gene expression real time PCR was performed. First, total RNA was isolated from the cells and 1 μg of the isolated RNA was reverse transcribed using random hexamer primers and MMLV (Moloney Murine Leukemia Virus) reverse transcriptase, according to manufacturer's instruction (Fermentas, Vilnius, Lithuania). The prepared cDNAs were amplified using TaqMan Universal PCR Master Mix (Perkin Elmer/Applied Biosystems Foster City, CA) according to the recommendations of the manufacturer in a total volume of 20 μl in an ABI PRISM 7500 Sequence Detection System (Applied Biosystems). Thermocycler conditions comprised an initial step at 95°C for 10 minute, which was followed by a 2-step PCR program at 95°C for 15 seconds and 60°C for 60 seconds for 40 cycles. Data were collected and quantitatively analyzed using SDS 2.1 software (Applied Biosystems). Rat β-actin gene was used as an endogenous control for sample normalization. Results were presented relative to the expression of β-actin. The PCR primers and probes detecting IFN-γ, IL-17, RorγT and β-actin were as follows: IFN-γ forward primer 5'-TGG CAT AGA TGT GGA AGA AAA GAG-3'; IFN-γ- reverse primer 5'-TGC AGG ATT TTC ATG TCA CCA T-3'; IFN-γ probe FAM 5'-TTT TGC CAG TTC CTC CAG ATA TCC AAG AAG A-3' TAMRA; IL-17 forward primer 5'-ATC AGG ACG CGC AAA CAT G-3'; IL-17 reverse primer 5'-TGA TCG CTG CTG CCT TCA C-3'; IL-17 probe FAM 5'-CTT CAT CTG TGT CTC TGA TGC TGT TGC TGC-3' TAMRA; RorγT forward primer 5'-GAC AGGG CCCC ACA GAG A-3'; RorγT reverse primer 5'-TTT GTG AGG TGT GGG TCT TCT TT-3'; RorγT probe: FAM 5'-CGA ACA TCT CGG GAG TTG CTG GCT-3' TAMRA; β-actin forward primer 5'-GCT TCT TTG CAG CTC CTT CGT-3'; β-actin reverse primer 5'-CCA GCG CAG CGA TAT CG-3'; β-actin probe VIC 5'-CAC CCG CCA CCA GTT CGC CAT-3' TAMRA. Accumulation of PCR products was detected in real time by monitoring the probe cleavage-induced mobilization of the reporter dye.

### ELISA and cell-based ELISA

Cells were cultivated for 3 h – 72 h as indicated in the Results section. Subsequently, cell culture supernatants were collected and cells pelleted by centrifugation (500 g, 3 min). Cell-free supernatants were frozen until analyzed by the protocol recommended by the manufacturers of the ELISA kits (OptEIA Mouse IL-17 Set, BD Biosciences; rat IFN-γ and rat IL-6 ELISA DuoSets, R&D systems, Minneapolis, MN). Cell-based ELISA (cELISA) was performed as described previously [18]. In brief, 4 × 10^5 ^cells were attached to plastic surface of 96-weel plates by poly-l-lysine coating. They were grown over-night in RPMI supplemented with 0.5% fetal calf serum, treated with MP for 2 hours and subsequently stimulated with ConA for 40 minutes. Finally, cells were fixed with paraformaldehyde and exposed to antibodies specific for p-ERK, p-p38, p-JNK, p-Jun and c-Fos, and corresponding secondary antibodies conjugated to horse-radish peroxidase (Santa Cruz Biotechnology, CA). Obtained values of absorbance were normalized to relative cell number, detected by crystal-violet staining.

### Cell proliferation assay

Cell proliferation was measured by incorporation of ^3^H-thymidine (Sigma) into DNA and by staining with carboxy-fluorescein diacetate succinimidyl ester (CFSE, Sigma). ^3^H-thymidine (5 μCi/ml) was added to cell cultures during last 16 h of 72 h incubation period of LNC. Its incorporation into cellular DNA, expressed as counts per minute (cpm), was determined in a scintillation counter. For CFSE staining LNC were exposed to 5 μM CFSE for 10 minutes, intensively washed and then cultivated for additional 48 hours. Subsequently, cells were subjected to flow cytofluorimetry and results analyzed with Cell Quest software (BD Biosciences).

### Statistical analysis

The results are presented as mean+/-SD of values obtained in a representative from at least three separate experiments with similar data. For EAE experiments, at least 15 rats per experiment were used, and the presented data were obtained from 3 or 4 rats per time point. Student's t test was performed for statistical analysis. A p value less than 0.05 was considered statistically significant.

## Results

### Methylprednisolone inhibits IL-17 and IFN-γ expression in mitogen-stimulated lymph node cells

**Figure 1:**
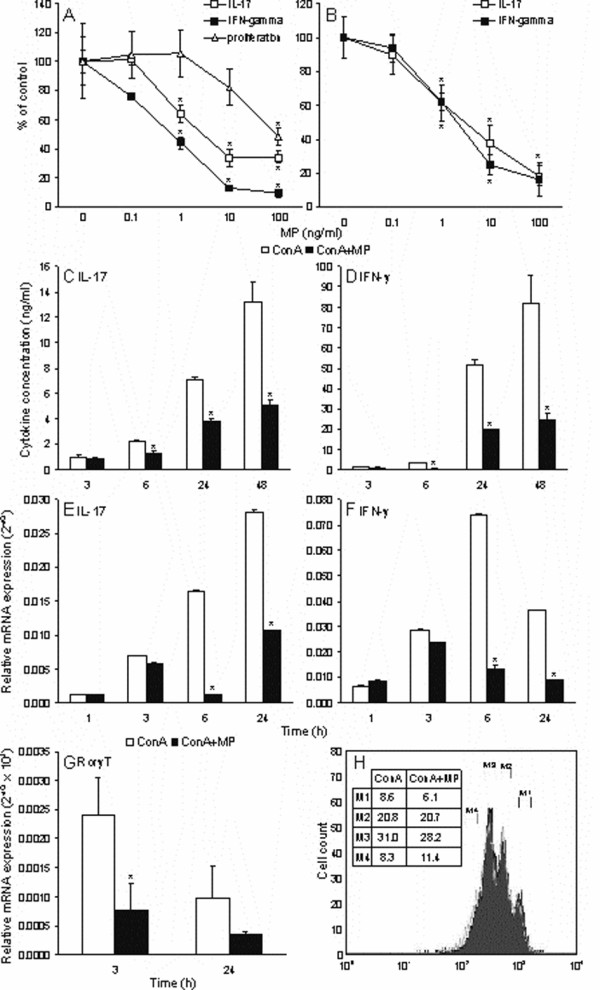
**The effect of MP on IL-17 and IFN-γ gene expression and production in LNC**. LNC isolated from DA rats (A, C-H) or AO rats (B) were stimulated with ConA (2.5 μg/ml) for 48 hours (A, B, E, F, H) or for various time periods (C, D) in the presence or absence of various MP concentrations (A, B), or with 10 ng/ml of MP (C-H). Subsequently, culture supernatants were collected for ELISA (A, B, C, D) or cells were collected for CFSE FACS analysis (H) or RNA isolation (E, F, G). Alternatively, cells were grown for additional 16 hours in the presence of ^3^H-thymidine for proliferation assay (A). Results are presented as % of values obtained in corresponding cultures without MP – control (A, B) or as concentrations (C, D), or as 2^-dCt ^(E, F, G). CFSE staining profiles are: dark grey shadow – ConA, light grey line – ConA+MP, while the numbers in the table represent % of cells in corresponding phase. *p < 0.05 represents statistically significant difference to the corresponding culture without MP.

Lymph node cells (LNC) isolated from DA rats were stimulated with concanavalin A (ConA, 2.5 μg/ml) for 48 hours in the presence or absence of various methylprednisolone (MP) concentrations (0.1 – 100 ng/ml). As a result, clear dose-dependent inhibition of IL-17 production was observed (Fig 1A). Similar results were obtained if LNC from AO rats were used (Fig 1B), thus excluding strain-specificity of the observed effect. In order to rule out that the observed effect of MP on cytokine production was a consequence of the restriction of cell proliferation or viability, ^3^H-thymidine incorporation assay, CFSE staining and trypan-blue exclusion test were performed. Although MP inhibited LNC proliferation in the highest dose (100 ng/ml), it did not significantly affect cell proliferation (Fig 1A, H), nor cell viability (data not shown) in doses equal or below 10 ng/ml. Consequently, MP of 10 ng/ml was used in the following experiments. The effect of MP was sustained throughout 48 hours of follow-up period, during which check-points were at 3 h, 6 h, 24 h, 48 h (Fig 1C). In an attempt to elucidate if MP inhibits the cytokines production through restriction of the gene expression, RT-PCR was conducted. Marked inhibitory effect of MP on the cytokines' gene expression induced by ConA stimulation was observed after 6 and 24 hours of incubation (Fig 1E, F). Thus, these results strongly suggested that MP potently inhibits both IL-17 and IFN-γ production through inhibition of mRNA generation. Importantly, MP also inhibited gene expression of the essential IL-17-promoting transcription factor RorγT (Fig 1G), thus suggesting that the observed inhibition of IL-17 production was, at least partly, mediated through down-regulation of RorγT expression.

### Methylprednisolone inhibits antigen-specific production of IL-17 and IFN-γ

**Figure 2:**
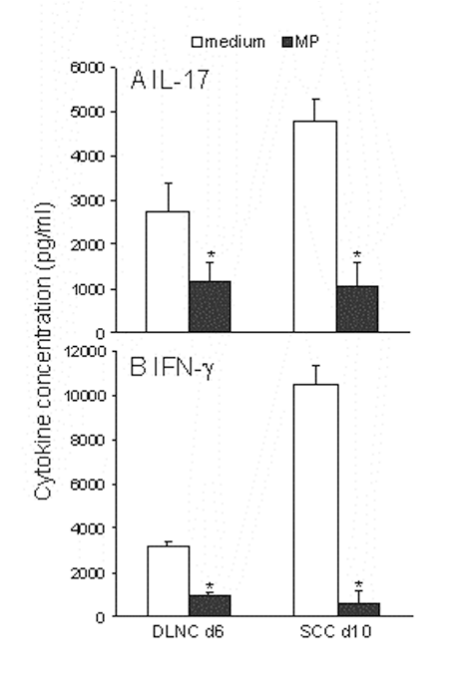
**The effect of MP on MBP-specific production of IL-17 and IFN-γ**. DLNC isolated from 4 rats 6 days after immunization with SCH-CFA, or SCC isolated from 3 rats 10 days after immunization with SCH-CFA (clinical score – 1.5) were stimulated with MBP (10 μg/ml) and cultivated in the presence or absence of MP (10 ng/ml) for 72 hours. *p < 0.05 represents statistically significant difference to the corresponding culture without MP.

As glucocorticoids are widely used in the treatment of CNS autoimmunity, we investigated the influence of MP on myelin basic protein (MBP)-induced IL-17 production. Cells for the investigation were isolated from draining lymph nodes (DLNC) of DA rats in the inductive phase of EAE (day 6 p.i.) or spinal cords (SCC) at the onset of the disease (day 10 p.i.). After isolation, both DLNC and SCC were cultivated for 72 hours with MBP (10 μg/ml) in the presence or absence of MP (10 ng/ml). As presented in Fig 2, release of IL-17 and IFN-γ from DLNC and SCC were markedly down-regulated in the presence of MP. Similar results were obtained if MP was applied to cultures that were not stimulated with MBP (spontaneous *ex vivo *release, data not shown), thus further fortifying the observation about the potency of MP in down-regulation of IL-17 and IFN-γ in lymphocytes from immunized rats. Together, these results clearly showed that MP inhibits antigen-induced production of IL-17, but once again, its effect on IFN-γ was more pronounced than on IL-17.

### Methylprednisolone inhibits IL-17 and IFN-γ production in T lymphocytes

**Figure 3:**
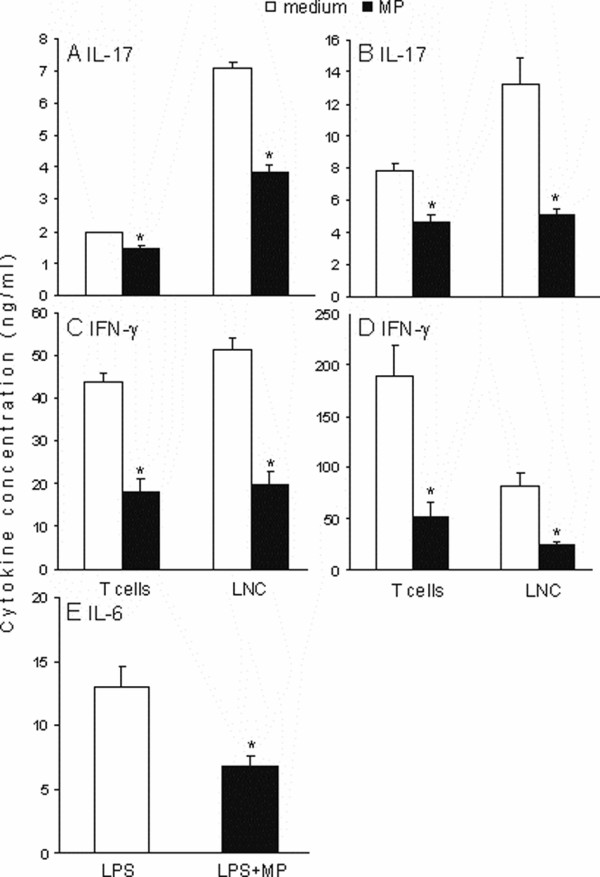
**The effect of MP on IL-17 and IFN-γ production in T lymphocytes**. CD3^+ ^cells and LNC were stimulated with anti-CD3 antibody and anti-CD28 antibody (both at 1 μg/ml) in the absence or presence of MP (10 ng/ml) for 24 (A, C) and 48 (B, D) hours, and cell culture supernatants analyzed for IL-17 (A, B) and IFN-γ (C, D). CD3^- ^cells were stimulated with LPS (1 μg/ml) for 48 hours and cell culture supernatants analyzed for IL-6 (E). Results are presented as % of values obtained in the corresponding cultures without MP. *p < 0.05 represents statistically significant difference to the corresponding culture without MP.

In order to investigate whether the observed inhibition of IL-17 production in LNC was a consequence of direct or indirect effect of MP on T cells, CD3^+ ^cells separation from LNC was performed. These cells were stimulated with anti-CD3 and anti-CD28 antibodies (both at 1 μg/ml) in the absence or presence of MP (10 ng/ml) for 24 and 48 hours, and cell culture supernatants were analyzed for the concentration of IL-17 and IFN-γ. As the result, MP inhibited IL-17 generation in purified T lymphocytes, but less efficiently than in LNC (Fig 3A, B), thus suggesting that MP affected IL-17 production in LNC through direct influence on T cells and indirect influence on accessory cells. Similarly to results obtained in LNC the effect of MP on IL-17 production by purified T lymphocytes was weaker than its influence on IFN-γ (Fig 3C, D). Interestingly, the extent of MP induced IFN-γ inhibition was similar in LNC and purified T cells, contrary to the difference observed in the effect of MP on IL-17 production by LNC and purified T cells (Fig 3A–D). In order to explore indirect influence of MP on IL-17 production, which could be important for the observed difference in efficiency of MP in inhibition of IL-17 in purified T cells and LNC, CD3^- ^cells were stimulated with LPS in the presence or absence of MP and production of a major IL-17-promoting cytokine – IL-6 was determined. As a result it was shown that MP potently inhibited IL-6 production in LPS-stimulated CD3^- ^cells, thus suggesting that indirect influence of MP on IL-17 production could be, at least partly, conducted through inhibition of IL-6 production by non-T cells.

### Methylprednisolone cooperates with IFN-γ in reduction of IL-17 generation in lymph node cells

**Figure 4:**
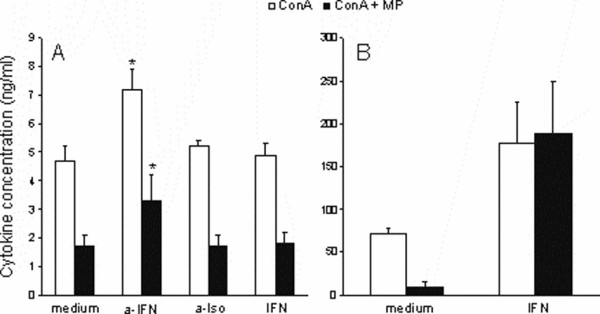
**The effect of IFN-γ addition or neutralization on the inhibition of IL-17 production by MP**. LNC were stimulated with ConA (2.5 μg/ml) and incubated with or without MP (10 ng/ml) and/or IFN-γ (IFN – 100 ng/ml), and/or anti-IFN-γ-neutralizing antibody (a-IFN – 1 μg/ml) or isotype control antibody (a-Iso – 1 μg/ml) for 24 hours. Subsequently, culture supernatants were collected for ELISA and IL-17 (A) and IFN-γ(B) production was determined. *p < 0.05 represents statistically significant difference to control cultures (medium).

Having in mind that IFN-γ is inhibitory factor for IL-17 production (3, 4), and that we observed almost complete reduction of IFN-γ, but not so extensive down-regulation of IL-17 by MP, our next step was to investigate if MP spared IL-17 generation through eliminating the other negative factor – IFN-γ. To that extent, LNC cultures stimulated with ConA and treated with MP were simultaneously stimulated with recombinant IFN-γ. However, there was no significant change in IL-17 production in the presence or absence of IFN-γ, irrespectively on the presence of MP (Fig 4A). In parallel IFN-γ production was also determined, and although it was obvious that MP had really pronounced effect on its production, the drug still did not abrogate it completely (Fig 4B). Therefore it was interesting to see what would happen if such remaining IFN-γ-production would be further abolished by the addition of neutralizing antibody specific for IFN-γ. As a result, such an antibody, but not the irrelevant isotype control antibody, up-regulated IL-17 production, both in the presence or absence of MP, thus suggesting that IFN-γ, even in a minute quantity that persisted after MP inhibition was a negative regulator of IL-17 in our experimental system, and that it cooperated with MP in reduction of IL-17 generation in LNC.

### Methylprednisolone inhibits Jun activation in ConA-stimulated lymph node cells

**Figure 5:**
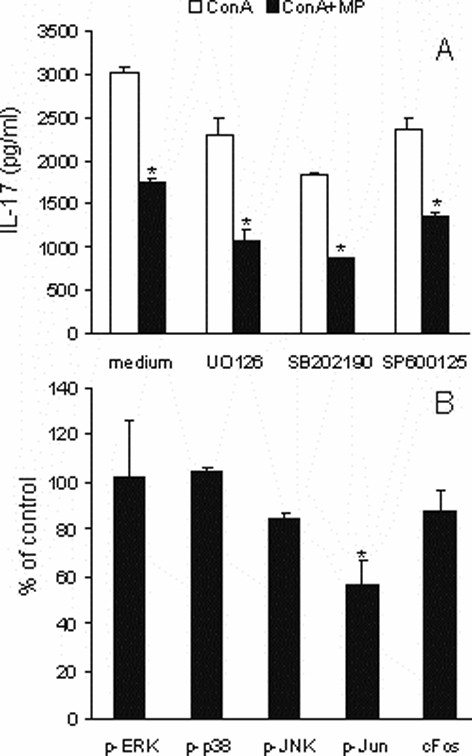
**The effect of MP on MAPK signaling in ConA-stimulated LNC**. A) LNC were stimulated with ConA (2.5 μg/ml) and incubated with or without MP (10 ng/ml) and/or ERK-inhibitor (UO126, 20 μM), or p38-inhibitor (SB202190, 20 μM), or Jnk-inhibitor (SP600125, 40 μM) for 24 hours. B) LNC cultivated with or without MP (10 ng/ml) for two hours and stimulated with ConA (2.5 μg/ml) for additional 40 minutes, were fixed and subjected to cELISA specific for phosphorylated ERK, p38, Jnk, Jun and c-Fos. cELISA results are presented as % of corresponding cultures without MP (control). *p < 0.05 represents statistically significant difference to the corresponding culture without MP.

Glucocorticoids bound to their receptors interfere with cytokines production acting directly on the gene expression or indirectly on the signal transduction. Therefore, the influence of MP on ConA-induced mitogen activated protein kinases (MAPK) signaling in LNC was investigated. LNC were stimulated with ConA and incubated with or without MP (10 ng/ml) and/or ERK-inhibitor (UO126, 20 μM), or p38-inhibitor (SB202190, 20 μM), or JNK-inhibitor (SP600125, 40 μM). Both MP and every of the signaling inhibitors used inhibited IL-17 production in ConA-stimulated LNC (Fig 5A). Moreover, if MP was combined with any of the inhibitors the inhibition was more pronounced, thus implicating that MP did not inhibit either ERK, or p38, or JNK signaling. Additionally, LNC pretreated with MP for two hours and subsequently stimulated with ConA for 40 minutes were analyzed for intracellular levels of phosphorylated ERK, p38 and JNK. As a result, MP did not inhibit activation of these crucial elements in MAPK signaling (Fig 5B). However, levels of phosphorylated Jun were markedly decreased in the presence of MP (Fig 5B), thus implying importance of transcription factor AP-1 (consisted of p-Jun and Fos) for the observed inhibitory effect of MP.

## Discussion

The present work is the first to show the efficiency of MP in inhibition of IL-17 expression in rat LNC. Interestingly, the effect of MP was less pronounced on IL-17 than on IFN-γ. The observed inhibition was, at least partly, conducted through inhibition of RorγT expression and activation of transcription factor AP-1 subunit – Jun.

Despite the evidence that some of the proinflammatory effects of IL-17 can be antagonized by GCs [19,20], just a few facts have been known about the influence of GCs on IL-17 production. In one report, methylprednisolone appeared to be only partially effective in blocking PMA/ionomycin-triggered IL-17 production *in vitro *by lymphocytes from healthy humans in comparison to extreme inhibitory action of Cyclosporin A in the same setting [20]. The influence of GCs on IL-17 expression *in vivo *was demonstrated in bronchial biopsy specimens of moderate-to-severe asthma patients by showing that the elevated number of IL-17 producing cells decreased to levels found in normal controls after oral treatment with GCs [21]. The present work accordingly proves that MP potently inhibits IL-17 expression and production in a strong, mitogen-stimulated T cell response, as well as in more subtle, antigen-specific response of T lymphocytes. However, the influence of MP is less effective if the drug is applied to purified T cells than to mixed population of LNC, thus suggesting that action of MP on IL-17 generation in rat LNC includes both direct influence on T cells and indirect influence on other LNC populations contributing to T cells IL-17 production. Indeed, the capability of GCs to affect activity of accessory LNC cells, including dendritic cells and macrophages, both at the level of membrane bound co-stimulatory molecules and cytokine production was previously described [12]. Production of numerous cytokines that have been shown important for the stimulation of IL-17 in T cells, such as IL-1, TNF-α, IL-6, IL-18 [22,23], could be affected in macrophages and dendritic cells by the influence of GCs [12,24]. In line with these data, we demonstrated that MP inhibited IL-6 production in LNC population devoid of T lymphocytes. It is thus expected that MP affects IL-17 production in LNC more potently than in purified T cells. Furthermore, among cells of LN there are other cell types, besides T cells, that could be relevant source of IL-17 [3], and that could contribute to the observed difference. However, the same phenomenon was not observed with IFN-γ, as MP had almost equal inhibitory effect on T cells as on LNC. Therefore, it seems that the direct effect of MP on T cells is crucial for IFN-γ inhibition, and although it was convincingly demonstrated that GK inhibit the production of IL-12, necessary for Th1 differentiation [12,24], it is tempting to speculate that additional influence through modulation of expression of molecules in accessory cells have minor contribution, if any. Although there are previous reports about the inhibitory effect of GCs on IFN-γ production in spleen cells [25], as well as on purified CD4^+ ^cells [16] in rats, this is for the first time that a comparative approach, including both starting population and purified T cells, in the investigation of the influence of GC on IFN-γ production is used. The potency of the direct effect of MP on IFN-γ production in T cells is supported by recent findings that GCs directly interfere with Tbet, the essential transcription factor of IFN-γ-producing cells [26]. Importantly, our results convincingly demonstrate that MP inhibits IFN-γ production more potently than IL-17 production, irrespectively of experimental setting used. Additionally, we also present evidence that small production of IFN-γ remained after MP action is still adequate to inhibit IL-17 production since the addition of anti-IFN-γ-neutralizing antibody eliminated such an inhibitory effect. Therefore, it seems that although MP inhibits IFN-γ, they still cooperate to limit IL-17 generation. The lack of deepening IL-17 inhibition by the addition of exogenous IFN-γ is unexpected but it might be explained by saturating effect of IFN-γ that remained upon MP treatment. It is on future investigations to explore if this complex *in vitro *relation is paralleled *in vivo*, and to find about its significance for the therapeutic efficiency of MP.

IL-17-producing cells are now considered to be the major culprits in various autoimmune disorders, including RA, IBD, MS, and/or their animal models, that were previously considered to be caused by IFN-γ-secreting Th1 cells [2,22,27]. Although neutralization or deletion of IFN-γ and/or molecules involved in IFN-γ production and effector functions paradoxically enhanced the autoimmunity in some experimental models [2,28], the role of this cytokine in organ-specific autoimmune diseases and its relationship to Th17 cells have not been fully understood, yet. A pivotal pathogenic role for IL-17 in the autoimmune response has been substantiated by attenuation of these and other disorders with IL-17 neutralization by anti-IL-17 antibodies or in mice genetically deficient for IL-17 or IL-17 receptor (IL-17R) [2,22,27]. First indications about importance of Th17 cells for the pathogenesis of autoimmune diseases in humans were elevated number of IL-17-producing cells and IL-17 in patients' circulation or at the sites of the autoimmune response and reduction of these parameters with immunomodulatory therapy [2,22,27]. Regarding MS, significant increase in IL-17 gene-expression and elevation of number of IL-17-producing CD4^+ ^and CD8^+ ^T cells in active lesions in comparison to silent lesions, or normal tissue were reported [29,30]. More direct evidence for the role of IL-17 in MS has recently been presented by Kebir et al., as they showed that human blood-brain barrier (BBB) endothelial cells in MS lesions express receptors for IL-17, and that IL-17 disrupts BBB tight junctions both *in vitro *and *in vivo *[31]. Th17 lymphocytes were also shown to transmigrate efficiently across BBB endothelial cells, highly express granzyme B, kill human neurons and promote CNS inflammation through CD4^+ ^lymphocyte recruitment [31]. Taken together, these recent data clearly suggest the importance of Th17 for the pathogenesis of MS. Our results suggest that a part of the efficiency of GCs in the therapy of MS, and other autoimmune diseases such as RA and IBD, is achieved through reduction of IL-17 generation. However, GCs are not absolutely efficient in the treatment of autoimmune disorders, as up to 30% of patients suffering from various autoimmune disorders do not respond adequately to the GC therapy [32]. In our experimental settings, IL-17 production is less sensitive than IFN-γ production to the influence of MP. Having in mind suggested importance of IL-17 and redundancy of IFN-γ for autoimmunity, one could speculate that the refractoriness to the therapy with GCs could be, at least partly, explained by the insufficient reduction of IL-17 production and possibly number and/or frequency of IL-17-producing cells. The idea is acceptable for MS, where it is known that the disease develops in various subjects as a consequence of different pathogenic mechanisms [33]. Again it is possible that Th17 response of some patients could be prevalent and those patients would therefore have weaker response to GC treatment. It has recently been reported that with patients refractory to GC therapy, there is a prevalence of special population of CD4^+^CD25^int ^cells among CD4^+ ^cells, and that this special sub-set is capable to proliferate in the presence of high dexamethasone concentrations [34]. Lee and co-authors propose a new paradigm for patients resistant to GC therapy, according to which GCs positively select CD4^+^CD25^int ^cells, thus generating a population of GC-resistant T cells that perpetuate ongoing inflammation. Additionally, it has recently been reported that in humans TGF-β potently restricts Th17 cells that produce IFN-γ, but not those that do not generate IFN-γ [35]. Taking into account these recent findings, it is tempting to speculate that GCs could also differentially affect IFN-γ-producing and non-producing Th17 cells, which could explain the overall difference in the efficiency of the effect of MP on IFN-γ and IL-17 in our system.

GCs are generally considered to exert their immunosuppressive effects through genomic effect, i.e. modulating activity of transcription factors [12,26,36]. Additionally, there has been an accumulating number of evidence suggesting non-genomic effects of GCs, exerted in signaling pathways up-stream of transcription factors [12,26,36]. In various experimental settings it has been shown that GCs are able to affect MAPK activation and function [12,26,36]. In our investigation, MP did not affect ERK and p38 activation in rat LNC, while it had only limited, statistically non-significant effect on JNK activation. Accordingly, inhibitors of all of the three MAPKs collaborated with MP in its action against IL-17 generation, thus suggesting that MP did not use any of the signaling routes for its effect on IL-17 production. Of course, the observed collaboration between MP and any of the inhibitors applied could also be a consequence of incomplete inactivation of the signaling pathways by these agents. However, we could not test such possibility as higher concentrations of MP or the inhibitors applied in our experiments affected cell viability. Still, for ERK and p38 the results of cELISA completely support results with inhibitors. However, situation with JNK is not as clear, especially as in the same setting MP potently inhibited Jun activation. This comes as a surprise, as it is presumed that Jun can be phosphorylated only by the action of JNK. Still, it was already reported that under the influence of dexamethasone the rate of decrease in JNK enzyme activity was more prominent than reduction in protein content [37]. Thus, in our case statistically insignificant reduction of p-JNK concentration in cells could result in significant down-regulation of Jun phosphorylation. Alternatively, there are also reports about JNK-independent activation of Jun [38,39] which would be supportive to hypothesis that in our case JNK-independent effect of MP on Jun activation took place.

## Conclusion

Our results add IL-17 to the list of cytokines production of which could be down-regulated by the influence of GCs. Additionally, we provide an interesting phenomenon of differential sensitivity of IL-17 and IFN-γ production to the influence of MP. Our ongoing research is thus dedicated to the influence of MP on IL-17 and IFN-γ generation *in vivo*, and includes subsequent investigation of the possible connection between resistance to GC-therapy and Th17 cells.

## Abbreviations

AO: albino oxford; APC: antigen presenting cell; CFA: complete Freund's adjuvant; CFSE: carboxy-fluorescein diacetate succinimidyl ester; CIA: collagen induced arthritis; CNS: central nervous system; ConA: concanavalin A; DA: dark agouti; DLNC: draining lymph node cells; EAE: experimental autoimmune encephalomyelitis; ERK: extracellular signal-regulated kinases; GC: glucocorticoid; G-CSF: granulocyte colony-stimulating factor; GR: glucocorticoid receptor; IBD: inflammatory bowel disease; IFN: interferon; IL: interleukin; JNK: Jun N-terminal kinase; LNC: lymph node cells; MBP: myelin basic protein; MHC: major histocompatibility complex; MMLV: moloney murine leukemia virus; MP: methylprednisolone; MS: multiple sclerosis; RA: rheumatoid athritis; SCC: spinal cord cells; SCH: spinal cord homogenate; TGF: transforming growth factor; Th: helper T cells; TNF: tumor necrosis factor.

## Authors' contributions

MMo, ŽM and ĐM carried out most of the experimental procedures and helped to draft the manuscript. DP, ES and MMa performed some of RT-PCR and cytofluorimetry experiments. ZR, ĐM, and MM–S conceived of the study, and participated in its design and coordination and helped to draft the manuscript. All authors read and approved the final manuscript.
